# Quantifying the Impact of Public Perceptions on Vaccine Acceptance Using Behavioral Economics

**DOI:** 10.3389/fpubh.2020.608852

**Published:** 2020-12-03

**Authors:** Steven R. Hursh, Justin C. Strickland, Lindsay P. Schwartz, Derek D. Reed

**Affiliations:** ^1^Applied Behavioral Biology Unit, Institutes for Behavior Resources, Baltimore, MD, United States; ^2^Department of Psychiatry and Behavioral Sciences, Johns Hopkins University School of Medicine, Baltimore, MD, United States; ^3^Department of Applied Behavioral Science, University of Kansas, Lawrence, KS, United States; ^4^Cofrin Logan Center for Addiction Research and Treatment, Lawrence, KS, United States

**Keywords:** COVID-19, vaccine, vaccination, demand, behavioral economics

## Abstract

This study was conducted to evaluate the impact of public perceptions of vaccine safety and efficacy on intent to seek COVID-19 vaccination using hypothetical vaccine acceptance scenarios. The behavioral economic methodology could be used to inform future public health vaccination campaigns designed to influence public perceptions and improve public acceptance of the vaccine. In June 2020, 534 respondents completed online validated behavioral economic procedures adapted to evaluate COVID-19 vaccine demand in relation to a hypothetical development process and efficacy. An exponential demand function was used to describe the proportion of participants accepting the vaccine at each efficacy. Linear mixed effect models evaluated development process and individual characteristic effects on minimum required vaccine efficacy required for vaccine acceptance. The rapid development process scenario increased the rate of decline in acceptance with reductions in efficacy. At 50% efficacy, 68.8% of respondents would seek the standard vaccine, and 58.8% would seek the rapid developed vaccine. Rapid vaccine development increased the minimum required efficacy for vaccine acceptance by over 9 percentage points, γ = 9.36, *p* < 0.001. Past-3-year flu vaccination, γ = −23.00, *p* < 0.001, and male respondents, γ = −4.98, *p* = 0.037, accepted lower efficacy. Respondents reporting greater conspiracy beliefs, γ = 0.39, *p* < 0.001, and political conservatism, γ = 0.32, *p* < 0.001, required higher efficacy. Male, γ = −4.43, *p* = 0.013, and more conservative, γ = −0.09, *p* = 0.039, respondents showed smaller changes in minimum required efficacy by development process. Information on the vaccine development process, vaccine efficacy, and individual differences impact the proportion of respondents reporting COVID-19 vaccination intentions. Behavioral economics provides an empirical method to estimate vaccine demand to target subpopulations resistant to vaccination.

## Introduction

A COVID-19 vaccine remains the most effective long-term solution to the COVID-19 pandemic. Vaccine safety and efficacy are of utmost importance, but do not guarantee uptake. Coverage rates required for COVID-19 herd immunity vary widely with estimates from 55 to 82% (1). When compared to influenza vaccination coverage, these required rates are high; for example, the 2018–19 adult influenza vaccination coverage was 45.3% (2). Achieving critical COVID-19 coverage will likely require a persuasive, evidence-based public health education campaign targeting misinformation and misperceptions about vaccines, generally, and a rapidly developed COVID-19 vaccine, in particular (3, 4).

Behavioral economics, a field applying behavioral science within economic frameworks, provides an empirical approach for public health officials to quantify attitudes toward COVID-19 vaccination. Behavioral economics is popular for its application of cognitive psychology to economic decision-making, helping to explain how cognitive biases and behavior can impact otherwise rational behavior (e.g., status-quo bias) (5–7). However, a less emphasized, but equally meritorious conceptual and research benefit of behavioral economics has been applying economic principles to behavior analysis or the “economics of behavior” (8). This area of behavioral economics uses economic concepts such as demand curves, open and closed economies, elasticity, and complementary and substitutable goods to help explain choice behavior (9–11).

Validated behavioral economic demand procedures known as *purchase tasks* developed within this tradition have been essential for advancing research across behavioral science by allowing for experimental evaluation of demand across a range of hypothetical circumstances. Purchase tasks require respondents to report their willingness to obtain a commodity at different prices, level of effort, or some other qualifying trade-off, and can provide a snapshot of consumption intentions under different market conditions. Such hypothetical purchase tasks simulate demand and significantly relate to or predict actual consumption (12, 13) or clinical measures associated with substance consumption (14, 15). Although such hypothetical tasks will never substitute for real, observed consumption, existing research suggests these are useful proxies when assessment of real-world behavior or other direct observation is impractical (16).

The ability to simulate contexts for which direct observation of consumption is impractical, impossible, or unethical renders purchase tasks a useful approach for estimating vaccine demand across circumstances such as messaging around vaccine development and expected efficacy and safety. Such modeling is relevant because these simulated purchase tasks provide an unambiguous and functional assessment of consumer decision-making. Moreover, these approaches permit experimental control and manipulation of market constraints—cost, risk, or access to alternatives—that are evaluated within the same individual responding to well-defined scenarios. Simulated purchase tasks provide incremental benefit above traditional discrete-choice, survey assessments (e.g., “Would you obtain a COVID-19 vaccine?”) for which differences in responding could be attributable to uncontrolled factors with substantively different predictions based on the specific mechanism impacted (e.g., between-person differences in expected price, efficacy, or safety of a vaccine). National estimates forecasting COVID-19 vaccination informed exclusively by single-item assessments, therefore, may grossly underestimate or overestimate vaccination coverage if variables like vaccine efficacy, development process, or safety are not comprehensively considered as part of vaccination projections [e.g., see (17)].

Existing behavioral economic research using simulated purchase tasks has shown them reflective of real-world behaviors in diverse public health arenas (e.g., sexual activity, substance use) (8, 14, 18). Behavioral economic procedures are particularly well-suited for forecasting intentions regarding consumption of novel commodities. Although such commodities may not yet be available, they can be described in simulated markets that can model demand for a hypothetical new product, medical treatment, or, in the immediate case, to-be-developed vaccines. Measurement of COVID-19 vaccination intentions within such a framework is rapid and expected to have clear public health ramifications given an ability to predict likely vaccination behavior under a variety of possible future scenarios and public perceptions linked to those scenarios. This study was conducted to use a hypothetical vaccine acceptance task, similar to a hypothetical purchase task, to evaluate the impact of public perceptions of vaccine efficacy on intent to seek COVID-19 vaccination. If the methodology demonstrates sensitivity to scenarios designed to emulated public perceptions, then this behavioral economic methodology could be used to inform future public health vaccination campaigns designed to influence public perceptions and improve public acceptance of the vaccine. This study specifically demonstrates how these methods can model the impact of key public perceptions, such as skepticism about the rigor of the vaccine development processes (3, 4, 19)—on intended COVID-19 vaccination to inform potential public health campaigns targeting vaccine coverage.

## Methods

### Sampling Procedure

Researchers recruited participants using the crowdsourcing platform Amazon Mechanical Turk (mTurk) from June 18–22, 2020. Crowdsourcing takes advantage of online “work-for-hire” pools for web-based research studies. Specifically, Amazon mTurk allows for sampling of participants willing to complete surveys online for compensation. This platform allows for easy access to a variety of demographics in geographically diverse areas (20, 21). For this study, participants meeting the specified inclusion/exclusion criteria could access recruitment information through mTurk and paid to complete the study materials online. Prior work has demonstrated the utility and validity of using crowdsourcing to sample participants for behavioral science research by showing correspondence in responding between crowdsourced samples and those recruited using traditional approaches (20, 21). Study recruitment required potential participants to be from the United States and have at least 100 previously approved tasks on mTurk with a 99% approval rating to complete the study. The University of Kansas IRB approved all procedures and participants reviewed an electronic informed consent form prior to participation. A total of 646 respondents completed study materials. One-hundred and twelve were removed for failing to provide systematic data on the purchase task procedure (e.g., reversals from zero) indicating failure to provide attentive and valid data. This resulted in an analyzed sample of 534 respondents.

### Vaccine Demand Procedure

A simulated purchase task procedure evaluated vaccine demand. Participants saw instructions to read vignettes describing a hypothetical situation in which they would have access to a developed COVID-19 vaccine (see instructions in Supplementary Material). The instructions indicated this vaccine would be the only COVID-19 vaccine available, that it was free of cost, would have to be administered now, and was approved by the FDA. Participants completed a series of confirmation questions to ensure they understood the language and premise of the vignette. Participants then accessed instructions to again read the vignette and report whether they would get the vaccine across a series of vaccine efficacies, which we operationally defined for participants as percentage reduction in COVID-19 hospitalization risk (100–0% effective in 10% increments). These tasks were intended to be hypothetical descriptions of vaccine development programs that modeled the zeitgeist of news coverage in April through June of 2020.

Two tasks were completed in a randomized order that manipulated the development process. In the first “Standard” vaccine development, participants received information indicating the vaccine was developed in a typical 18-month vaccination process.

“Suppose a COVID-19 vaccine was developed in a total of 18 months, with delivery to the general population by July 2021. **Imagine the vaccine has been approved by the Food and Drug Administration (FDA) and the vaccine has undergone a standard and rigorous vaccine evaluation**. This evaluation included all three phases of human clinical trials to determine the vaccine's safety and effectiveness. You can get the vaccine through your doctor, at no cost to you.”

In the second “Warp Speed” vaccine development, participants received information that the vaccine was developed in an expedited 6-month “Operation Warp Speed,” similar to the program introduced in the US in April 2020 (22).

“Suppose a COVID-19 vaccine was developed in a total of 6 months, with delivery to the general population by November 2020. Imagine the vaccine has been approved by the Food and Drug Administration (FDA) as part of an accelerated partnership between the FDA, Centers for Disease Control (CDC), and pharmaceutical companies (this effort is called Operation Warp Speed). The planned partnership will develop a collaborative framework for prioritizing vaccine and drug candidates, streamlining clinical trials, coordinating regulatory processes, and/or leveraging assets among all partners to rapidly respond to the COVID-19 and future pandemics. **The FDA has relaxed some of its strict evaluation criteria to get the vaccine to the public quickly, but this vaccine will still be approved by the FDA.”**

### Health History and Participant Covariates

Following completion of both vaccine demand tasks, respondents completed the Generic Conspiracist Beliefs scale (GCB) (23) and the Social and Economics Conservatism Scale (SECS) (24) to measure conspiracy beliefs and social and economic conservatism, respectively. On both scales, higher values indicate more conspiracy or conservative beliefs, respectively. Respondents also completed measures of vaccine history, COVID-19 prevention behaviors, and demographics.

### Data Analysis

We computed aggregate demand curves to model estimated vaccine coverage. An exponential demand function (Equation 1) (25) was fit to the proportion of participants accepting vaccination at each efficacy. To account for well-established psychophysical scaling of risk perceptions, vaccine efficacy percent was converted to odds against efficacy (θ). The aggregate demand, *Q*, was modeled with the following exponential equation with odds against efficacy (θ) controlling the rate of decline in acceptance.

(1) $$\begin{array}{r} \log\left(Q\right)=\log\left(Q_{0}\right)+k\left(e^{-\alpha Q_{0}\theta }-1\right) \end{array}$$

The maximum (*Q*_0_) was constrained to the maximum observed acceptance at 100% efficacy (θ = 0), with the constant *k* (span of % vaccine acceptance in log units) and α (rate of change in demand elasticity) parameters unconstrained and fit in the exponential model. Applying the model to the data allowed us to calculate predicted levels of acceptance at each efficacy point for each vaccine scenario. The model has two free parameters and it fit to 9 data points (10-point increments from 10 to 90%), making it unlikely for the model to overfit the data.

Minimum required efficacy for each vaccine task served as a within-subject measure and calculated as the individual median value between last accepted and first rejected vaccine efficacy. Higher minimum required efficacy values are indicative of a need for higher vaccine efficacy for vaccine intention. Linear mixed effect models evaluated minimum required efficacy outcomes for: (1) development process effects, (2) main effects of individual characteristics, and (3) interactions between development process and individual characteristics. Fixed effect estimates (γ) for the main effect models were interpreted as the change in the minimum required efficacy value with a one-unit increase (continuous variables) or group membership (dichotomous variables). Interaction model fixed effect values reflected the interaction between the effect of rapid vaccine development framing (Level 1 variable) and the individual characteristic (Level 2 variable). Main effect models and interaction models were conducted separately, but each contained all fixed effects in a single multivariable model. All models included a random effect term (random slope) for the rapid vaccine development framing parameter and a random intercept term. All analyses were conducted in *R* using two-tailed tests and a type I error rate of 0.05.

## Results

### Sample Demographics

Respondents reported an average age of 41.9 years old (SD = 13.4), were predominately White (77.2%), and approximately half indicated male gender (49%). About a fifth endorsed the belief that vaccines cause autism (21.0%) and 58.3% reported receiving a flu vaccine in the past 3 years. At the time of the assessment, 58.1% reported always practicing social distancing and 56.6% reported always using a face mask. Participants scored an average of 38.2 (SD = 14.9) on the GCB scale and 70.4 (SD = 21.7) on the SECS.

### Vaccine Demand by Development Process and Covariates

**Figure 1:**
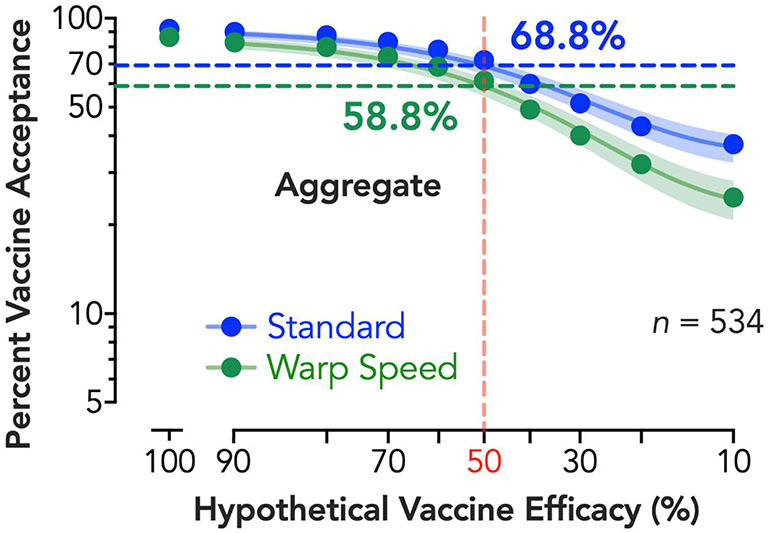
Estimated percent vaccine acceptance by efficacy and vaccine development process. Vertical reference line (shaded red) depicts the FDA's 50% vaccine efficacy target, with horizontal lines depicting the vaccine acceptance associated with 50% efficacy for both standard (shaded blue) and expedited (shaded green) development processes; adjacent numerical values indicate exact solutions for coverage at 50% per group (shaded, respectively). Shaded 95% confidence bands were generated using bias-corrected and accelerated (BCa) bootstrapped values based on 10,000 iterations. A corrected (for small numbers of data points) Akaike information criterion test indicates a >99.99% probability the curves are best described independently (i.e., do not share best fit parameters in the nonlinear curve-fitting).

Vaccine demand decreased systematically with reduced expected vaccine efficacy. The exponential model described aggregate demand well (*R*^2^ > 0.99). Evaluation of demand curves indicated greater reductions in vaccine demand by efficacy under Operation Warp Speed and permitted estimated vaccine coverage at critical threshold targets [e.g., 50% efficacy of vaccination (26); Figure 1]. At 50% efficacy, 68.8% of respondents would seek the vaccine if developed under standard procedures, and acceptance drops by 10–58.8% if vaccine was described as developed under the expedited “Warp Speed” process. At an individual level, rapid vaccine development increased minimum required efficacy for vaccination by over 9% points, γ = 9.36, *p* < 0.001.

**Figure 2:**
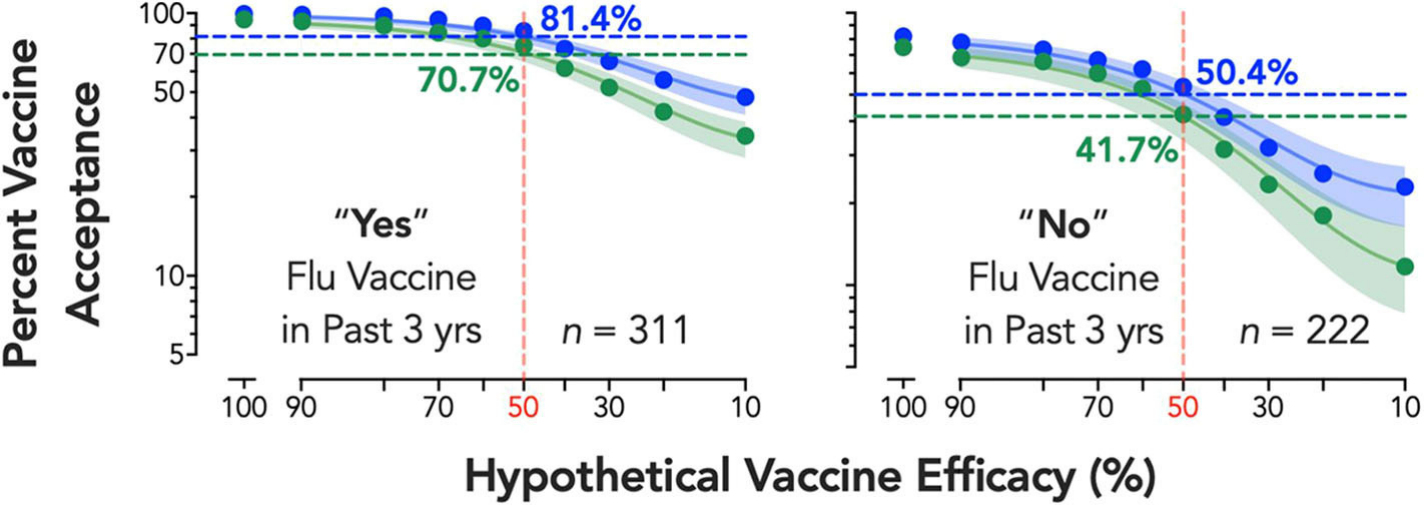
Estimated percent vaccine acceptance by efficacy and vaccine development process for flu vaccine subgroup. Respondents reporting “Yes” (left panel) or “No” (right panel) to a question on whether they received the seasonal flu vaccine at least once in the past three years (data on seasonal flu vaccine receipt from one respondent was missing). Vertical reference lines (shaded red) depict the FDA's 50% vaccine efficacy target, with horizontal lines depicting the vaccine acceptance associated with 50% efficacy for both standard (shaded blue) and expedited (shaded green) development processes; adjacent numerical values indicate exact solutions for coverage at 50% per group (shaded respectively). Shaded 95% confidence bands were generated using bias-corrected and accelerated (BCa) bootstrapped values based on 10,000 iterations. A corrected (for small numbers of data points) Akaike information criterion test indicates a >99.99% probability the curves are best described independently (i.e., do not share best fit parameters in the nonlinear curve-fitting).

The main effects of individual characteristics and interaction effects are shown in Table 1. Participants reporting past-3-year flu vaccination, γ = −23.00, *p* < 0.001, and male respondents, γ = −4.98, *p* = 0.037, accepted lower efficacy, while respondents reporting greater conspiracy beliefs, γ = 0.39, *p* < 0.001, and political conservatism, γ = 0.32, *p* < 0.001, required higher vaccine efficacy. Vaccine development process also interacted with gender, γ = −4.43, *p* = 0.013, and political conservatism, γ = −0.09, *p* = 0.039, with male and more conservative respondents showing smaller changes in minimum required efficacy by development process. Significant individual variables translated to substantive shifts in estimated vaccine intentions at 50% efficacy—for example, 70.7% vaccine acceptance vs. 41.7% acceptance for an expedited vaccine in flu vs. non-flu vaccine utilizers, respectively (Figure 2). For the standard vaccine process, acceptance was over 81% for flu vaccine utilizers and only 50.4% for non-flu vaccine utilizers.

**Table 1:** Linear mixed effect multivariable models for individual minimum required vaccine efficacy.

	**Main Effect Model**		**Interaction Model**	
	**γ (95% CI)**	***p***	**γ (95% CI)**	***p***
**Vaccine Development**				
Rapid vaccine development	**9.36 (7.64, 11.09)**	**<0.001**	–	–
**Vaccine use and conspiracy beliefs**				
Past 3 year flu vaccination	**−23.00 (−27.73**, **−18.27)**	** <0.001**	0.66 (−2.86, 4.19)	0.715
Vaccines (MMR) causes autism	0.69 (−5.38, 6.75)	0.825	−0.74 (−5.25, 3.78)	0.752
GCB conspiracy scale	**0.39 (0.22, 0.56)**	** <0.001**	−0.10 (−0.23, 0.02)	0.112
**COVID-19 Behaviors and Beliefs**				
Consistent mask use	−2.63 (−8.32, 3.06)	0.367	4.00 (−0.24, 8.24)	0.067
Consistent social distancing	−0.17 (−5.70, 5.36)	0.953	−1.52 (−5.64, 2.60)	0.475
Expected community vaccination	**−0.20 (−0.31**, **−0.08)**	** <0.001**	−0.03 (−0.11, 0.06)	0.546
**Demographics**				
Age	0.13 (−0.06, 0.32)	0.196	0.12 (−0.02, 0.26)	0.095
Gender	**−4.98 (−9.62**, **−0.33)**	**0.037**	**−4.43 (−7.89**, **−0.97)**	**0.013**
Race	−0.55 (−3.51, 2.40)	0.714	−1.57 (−5.76, 2.62)	0.466
Subjective socioeconomic status	−2.67 (−8.29, 2.95)	0.354	0.78 (−1.43, 2.98)	0.495
SECS (political conservatism)	**0.32 (0.20, 0.44)**	** <0.001**	**−0.09 (−0.18**, **−0.01)**	**0.039**

*Models included 526 respondents (eight removed for missing values on model variables). GCB, Generic Conspiracist Beliefs (higher values are greater conspiracy beliefs); SECS, Social and Economic Conservatism Scale (higher values are greater conservative beliefs). Subjective socioeconomic status evaluated as (0, hard time buying the things needed: 1, just enough money; 2, no problem buying things and sometimes buy special things; 3, enough money to buy pretty much anything wanted). Categorical coding reference (REF) group: Past 3 Year Flu Vaccine (REF = No); Vaccines (MMR) Causes Autism (REF = No); Consistent Mask Use (REF = Less than Always); Consistent Social Distancing (REF = Less than Always); Gender (REF = Female); Race (REF = White). Bold, statistically significant*.

### Estimated Vaccine Coverage

**Figure 3:**
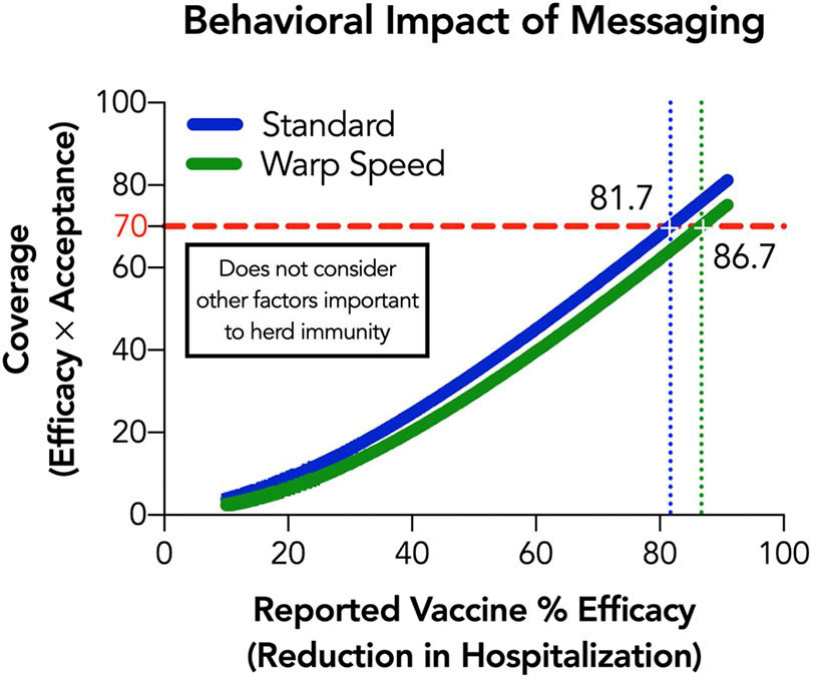
Estimated vaccine coverage by efficacy and vaccine development process. Horizontal reference lines (red) indicate a 70% coverage level and vertical reverence lines depict the vaccine efficacy associated with 70% coverage for both standard (shaded blue) and expedited (shaded green) development processes; adjacent numerical values indicate exact solutions for efficacy at 70% coverage per group.

Demand curve modeling can help better estimate total vaccine coverage by modeling demand for vaccines across every potential efficacy. To illustrate this, we calculated estimated vaccine coverage at the proposed vaccine efficacy multiplied by the estimated vaccine demand (acceptance). Figure 3 shows the estimated vaccine coverage for both the standard and Warp Speed vaccines. Coverage rates are compared to a 70% coverage, which is an estimate of the level of immunity required in a population, depending on the rate of virus transmission (1, 27). To achieve a vaccine coverage rate of 70%, advertised vaccine efficacy would need to be at least 81.7% for the standard vaccine and 86.7% for the Warp Speed vaccine.

## Discussion

Concrete data relating individual factors and vaccine messaging to expected COVID-19 vaccination are critical for designing effective public health campaigns. Here, we describe a rapid-to-implement, theory-based, and actionable method for detecting personal and structural variables promoting vaccine intentions toward the goal of identifying optimal dissemination tactics for swift deployment once a vaccine is developed and marketed as an outcome of either standard or “Operation Warp Speed” processes.

A sizable reduction in vaccination intentions was observed for a vaccine developed under an “Operation Warp Speed” process. This finding is consistent with scientific commentaries (3) and interviews with key public officials emphasizing potential ramifications of messaging around this program (e.g., from NIAID director Dr. Anthony Fauci, “I really don't like the word Warp Speed, because what it does is it implies carelessness in stepping over important steps”) (28). Despite the fact that Dr. Fauci, among others, indicated safety would not be compromised, there was general concern in the public sphere about Operation Warp Speed due to lack of educational campaigns at that time. Multiple news sources indicated an accelerated vaccine might have safety concerns, with headlines like “Trump Seeks Push to Speed Coronavirus Vaccine, Despite Safety Concerns,” (29) and “Trump's “Operation Warp Speed” Aims to Accelerate Vaccine Development Against Adviser's Warnings” (30). The vignettes for this study were created in May 2020 and the language reflected information from NIH press releases from late April 2020 about the intention of Operation Warp Speed (22) as well as the sentiment of news coverage during that time. At that time, the public had not yet seen detailed descriptions of Operation Warp Speed, and there was public concern about whether the vaccines would be safe and effective.

The purpose of this study was to assess how framing of the vaccine question would affect willingness to get vaccinated. Concerns surrounding the public perception of vaccination safety are important even if the framing of the question is not a completely accurate description of the Operation Warp Speed program. The current findings corroborate these concerns by demonstrating an approximate 9-point increase in necessary efficacy to promote vaccination under an expedited process. Such findings do not diminish possible benefits of an expedited process, but instead indicate that vaccination campaigns will likely require solutions targeting cognitive biases to effectively address false beliefs and skepticism about the rigor of vaccine development (4, 31). Evaluation of individual differences in vaccination intentions also highlights key factors and targets for public health efforts to promote vaccination, like choice strategies bundling flu and COVID-19 vaccines to facilitate vaccination rates among vaccine seekers or targeted campaigns addressing conspiracy beliefs. The key take-away of this demonstration is that the perception an individual has of the vaccine development process can impact their willingness to get the vaccine, and that impact can be quantified. However, it is important to make a distinction between the public perceptions emulated in this study using hypothetical scenarios, and the actual vaccine acceptance rates for any vaccine developed via Operation Warp Speed or any other program that may be coupled with publicity to support public confidence in the safety and efficacy of the vaccines.

Although vaccine efficacy impacted behavioral demand for vaccines as reported here, it also impacts biological immunity of vaccinated individuals. An advertised and achieved vaccine efficacy may, therefore, have a multiplicative effect on total vaccine coverage (the percentage of the population vaccinated and immune)—put simply, understanding impacts of how a reported efficacy impacts biological immunity and willingness to get the vaccine is a critical public health effort. This methodology could therefore be used to estimate overall vaccine effectiveness in the projected population. Rapid vaccine demand determinations using methods like those described here allow for easy resampling of vaccine intentions over time and more accurate determinations of estimated vaccine coverage rates across the population.

Using this methodology, evidence robustly supports relations between simulated demand assessments and concurrent and prospective prediction of real-world public health behaviors (8, 14, 18). However, limitations of the current study include non-probability sampling and assessment of intentions and self-reported histories, rather than actual vaccinations; note, however, prior evidence has shown a correspondence between seasonal influenza vaccination and pandemic influenza vaccination (32). The within-subject assessment is a notable strength as it afforded opportunities to evaluate person-level factors related to vaccination, overall, and the impact of development process messaging, specifically. Results show that age, race, and socioeconomic status did not impact general intention to receive a vaccination or the interaction with the framing condition. Although these person-level factors may be eventual barriers to actual vaccination, they did not affect vaccination intentions in this study, possibly because the sampling method may not have adequately represented a sufficient range of these key demographics.

The broad economic impact of the COVID-19 pandemic amplifies the need for rapid initiatives to promote herd immunity using novel COVID-19 vaccination. An essential ingredient in that initiative is human behavior to accept a vaccine. As demonstrated here, behavioral economics provides a key scientific framework to assess and guide public health messaging to ultimately increase vaccine coverage.

## Data Availability Statement

The raw data supporting the conclusions of this article will be made available by the authors, without undue reservation. Requests to access the datasets should be directed to Derek D. Reed, dreed@ku.edu.

## Ethics Statement

The studies involving human participants were reviewed and approved by The University of Kansas IRB. Written informed consent was not provided because electronic informed consent was provided and approved by the ethics committee.

## Author Contributions

SH, JS, LS, and DR contributed to the conception, design of the study and materials, interpreted the results and wrote the manuscript. JS and DR analyzed the data. All authors contributed to the article and approved the submitted version.

## Conflict of Interest

The authors declare that the research was conducted in the absence of any commercial or financial relationships that could be construed as a potential conflict of interest.
