# Supplementary material for: Quantifying the Impact of Public Perceptions on Vaccine Acceptance Using Behavioral Economics
Source: Front Public Health. 2020 Dec 3;8:608852. doi: 10.3389/fpubh.2020.608852 (PMC7744757; doi:10.3389/fpubh.2020.608852)
Supplement: Supplementary file 1 [file Data_Sheet_1.docx]

**Supplemental Materials**

**Study Vignettes**

**Operation “Warp Speed” Vaccine Development**

Please read and consider the following.

Vaccines provide immunity to a disease by developing antibodies to fight it – this means that while you may still get COVID-19 if vaccinated, the vaccine can reduce the chance of hospitalization at different levels of vaccine efficacy. You can get the vaccine through your doctor, at no cost to you.  

Suppose a COVID-19 vaccine was developed in a total of 6 months, with delivery to the general population by November 2020. Imagine the vaccine has been approved by the Food and Drug Administration (FDA) as part of an accelerated partnership between the FDA, Centers for Disease Control (CDC), and pharmaceutical companies (this effort is called Operation Warp Speed). The planned partnership will develop a collaborative framework for prioritizing vaccine and drug candidates, streamlining clinical trials, coordinating regulatory processes and/or leveraging assets among all partners to rapidly respond to the COVID-19 and future pandemics.  **The FDA has relaxed some of its strict evaluation criteria to get the vaccine to the public quickly, but this vaccine will still be approved by the FDA.**
Assumptions:

- The vaccine is easily administered by a doctor
- The vaccine is available to you without cost (free)
- You have the same income/savings as you do now
- You have no access to any other vaccines available for COVID
- The vaccine must be administered at the time of receiving it (you can’t save it to use at a later date)
- This vaccine must only be used for you (you cannot use this vaccine for friends or family members)
- This vaccine is approved by the FDA

Remember that there are no “right” or “wrong” answers. Please respond honestly, as if you were actually in this scenario.

Given the above scenario, please indicate whether or not you would you get the COVID-19 vaccine if the pharmaceutical company claims it reduced chances of hospitalization by each of the following percentages (X%):

**Rigorous Vaccine Development**

Please read and consider the following.

Vaccines provide immunity to a disease by developing antibodies to fight it – this means that while you may still get COVID-19 if vaccinated, the vaccine can reduce the chance of hospitalization at different levels of vaccine efficacy. You can get the vaccine through your doctor, at no cost to you.  

Suppose a COVID-19 vaccine was developed in a total of 18 months, with delivery to the general population by July 2021. **Imagine the vaccine has been approved by the Food and Drug Administration (FDA) and the vaccine has undergone a standard and rigorous vaccine evaluation.** This evaluation included all 3 phases of human clinical trials to determine the vaccine’s safety and effectiveness. You can get the vaccine through your doctor, at no cost to you.  


Assumptions:

- The vaccine is easily administered
- The vaccine is available to you without cost (free)
- You have the same income/savings as you do now
- You have no access to any other vaccines available for COVID
- The vaccine must be administered at the time of receiving it (you can’t save it to use at a later date)
- This vaccine must only be used for you (you cannot use this vaccine for friends or family members)
- This vaccine is approved by the FDA

Remember that there are no “right” or “wrong” answers. Please respond honestly, as if you were actually in this scenario.

Given the above scenario, please indicate whether or not you would you get the COVID-19 vaccine if the pharmaceutical company claims it reduced chances of hospitalization by each of the following percentages (X%):

**Response Options**

**
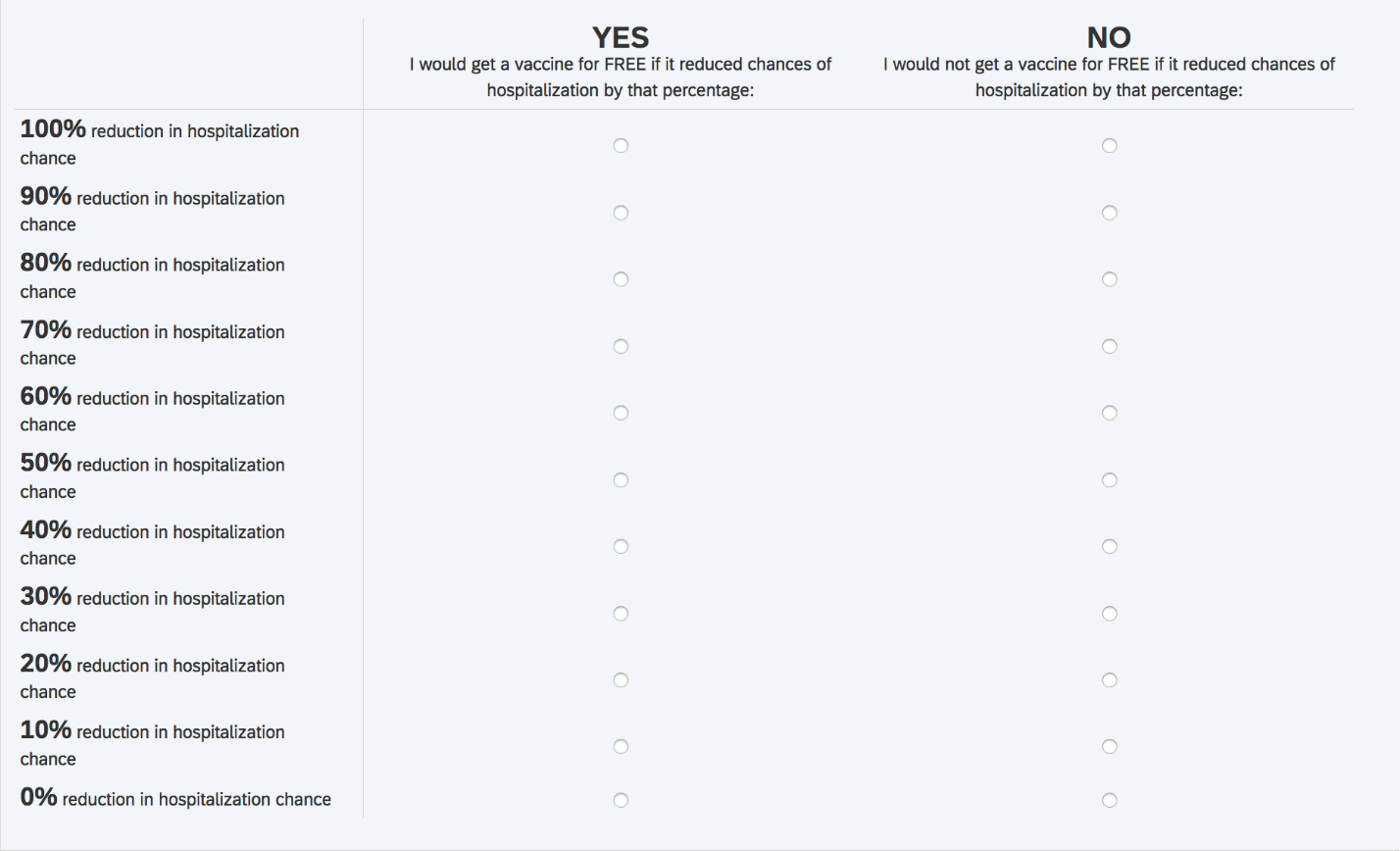
**
